# Self-reported leisure time physical activity: a useful assessment tool in everyday health care

**DOI:** 10.1186/1471-2458-12-693

**Published:** 2012-08-24

**Authors:** Lars Rödjer, Ingibjörg H Jonsdottir, Annika Rosengren, Lena Björck, Gunnar Grimby, Dag S Thelle, Georgios Lappas, Mats Börjesson

**Affiliations:** 1 Department of Emergency and Cardiovascular Medicine, The Sahlgrenska Academy, University of Gothenburg, Gothenburg, Sweden; 2 Department of Medicine, Halland Hospital Varberg, Träslövsvägen 68, Varberg, SE 432 81, Sweden; 3 Institute of Stress Medicine, Gothenburg, Sweden; 4 Department of Neuroscience and Physiology, Section of Clinical Neuroscience and Rehabilitation, The Sahlgrenska Academy, University of Gothenburg, Gothenburg, Sweden; 5 Department of Biostatistics, Institute of Basic Medical Sciences, Oslo University, Oslo, Norway

## Abstract

**Background:**

The individual physical activity level is an independent risk factor for cardiovascular disease and death, as well as a possible target for improving health outcome. However, today´s widely adopted risk score charts, typically do not include the level of physical activity. There is a need for a simple risk assessment tool, which includes a reliable assessment of the level of physical activity. The aim of this study was therefore, to analyse the association between the self-reported levels of physical activity, according to the Saltin-Grimby Physical Activity Level Scale (SGPALS) question, and cardiovascular risk factors, specifically focusing on the group of individuals with the lowest level of self-reported PA.

**Methods:**

We used cross sectional data from the Intergene study, a random sample of inhabitants from the western part of Sweden, totalling 3588 (1685 men and 1903 women, mean age 52 and 51). Metabolic measurements, including serum-cholesterol, serum-triglycerides, fasting plasma-glucose, waist circumference, blood pressure and resting heart rate, as well as smoking and self-reported stress were related to the self-reported physical activity level, according to the modernized version of the SGPALS 4-level scale.

**Results:**

There was a strong negative association between the self-reported physical activity level, and smoking, weight, waist circumference, resting heart rate, as well as to the levels of fasting plasma-glucose, serum-triglycerides, low-density lipoproteins (LDL), and self-reported stress and a positive association with the levels of high-density lipoproteins (HDL). The individuals reporting the lowest level of PA (SGPALS, level 1) had the highest odds-ratios (OR) for having pre-defined levels of abnormal risk factors, such as being overweight (men OR 2.19, 95% CI: 1.51-3.19; women OR 2.57, 95 % CI: 1.78-3.73), having an increased waist circumference (men OR 3.76, 95 % CI: 2.61-5.43; women OR 2.91, 95% CI: 1.94-4.35) and for reporting stress (men OR 3.59, 95 % CI: 2.34-5.49; women OR 1.25, 95% CI: 0.79-1.98), compared to the most active individuals, but also showed increased OR for most other risk factors analyzed above.

**Conclusion:**

The self-reported PA-level according to the modernized Saltin-Grimby Physical Activity Level Scale, SGPALS, is associated with the presence of many cardiovascular risk factors, with the most inactive individuals having the highest risk factor profile, including self-reported stress. We propose that the present SGPALS may be used as an additional, simple tool in a routine risk assessment in e.g. primary care, to identify inactive individuals, with a higher risk profile.

## Background

Physical activity (PA) is defined as any bodily movement produced by the skeletal muscles, resulting in increased energy expenditure [1]. Regular PA is associated with a lower risk of cardiovascular and overall morbidity and mortality [2]. Indeed, physical inactivity was recently ranked as the fourth most important risk factor of mortality worldwide, by the World Health Organization (WHO) [3]. The recommended level of medium intensity aerobic activity being 150 minutes/week, only half of the adult population in the USA reach this level of PA according to self-reported data [4], while the Eurobarometer study estimated that only 23 % of the Swedish population were sufficiently physically active [5].

Both cardiorespiratory fitness (CRF), defined as the maximum oxygen uptake on treadmill or by bicycle ergometer [1], and the level of physical activity have been found to be independently associated with cardiovascular risk factors, such as blood lipids, body mass index (BMI), blood pressure and insulin resistance [6,7], as well as predicting morbidity and mortality [8,9]. In addition, sedentary behaviour and few breaks in sitting time, is also independently associated to cardiovascular risk factors [10]. In addition to the direct effects on the cardiovascular system, physical inactivity also increases future risk of developing mental disorders and stress [11].

The definitions of sedentary behaviour has varied in the literature, initially including a low physical activity level in addition to being still, making comparisons between studies difficult [12]. The health risk related to sedentary behaviour seems to be independent of the time spent performing light and moderate to vigorous PA [13,14]. In these studies, sedentary behaviour was defined as an accelerometer count of <100/min, but has also been defined as all activities that consume not over 1.0-1.5 metabolic equivalent units (METs; equivalent to energy expenditure from oxygen uptake of 3.5 ml·kg^-1^·min^-1^) [15]. Recently the Sedentary Behaviour Research Network has suggested a standardized description [16], defining inactive individuals are those not meeting recommended level of PA, while sedentary behaviour is defined as any awake activities while sitting or reclining posture with an energy expenditure <1.5 METs. Moreover the actual sitting time has recently also been independently related to increased risk for all-cause and cardiovascular disease (CVD) mortality [17,18].

Indeed, the greatest net-effect for health may be gained by any positive change from sedentary behavior [19]. As a result, identifying and targeting physically inactive, including sedentary, or insufficiently active individuals, is one of the major tasks in primary and secondary prevention in everyday health care.

The Framingham Risk Score and the European Society of Cardiology’s SCORE, based on cholesterol, gender, age, blood pressure and smoking, are used to identify individuals with increased risk of cardiovascular events [20,21]. However, both risk score charts lack information about the physical activity level, which would be a valuable contribution to identify patients at risk, as both physical activity levels and CRF are important independent risk factors for CVD [9,22]. While direct measurement of the individual CRF level, using e.g. bicycle ergometers or treadmill tests may not be feasible in everyday health care, a simple assessment of the physical activity level, identifying patients at potentially increased cardiovascular risk, by being physically inactive, would be useful.

In a previous longitudinal study, we used a four level scale to assess the self-reported physical activity level in a working population, showing an association with both perceived stress and self-reported mental disorders [11]. This scale was originally developed by Saltin and Grimby (here termed Saltin-Grimby Physical Activity Level Scale, SGPALS) [23].

In the present study, we thus aimed to study the relationship between the self-reported level of PA according to the modernized version of SGPALS and other traditional cardiovascular risk factors in a large Swedish population. We also wanted to characterise the subjects having the lowest level of PA, according to SGPALS, with regard to their risk of having pre-defined abnormal levels of the classical risk factors.

## Methods

This study is a part of the Intergene study, which was conducted between April 2001 and December 2004, and included a random sample of inhabitants of Region Västra Götaland from central population registry, of both sexes, aged between 25 and 74 years. In total, 8820 individuals were initially selected to participate of whom 194 were not eligible, due to either having an unknown address, change of address or having died prior to contact. The survey response-rate was 44 % (women) and 39 % (men), and a total of 3610 individuals (1908 women/1702 men), with a mean age of 52 (women) and 51 years (men), were finally included in the study. The question regarding PA level was answered by 1903 women and 1685 men.

The aim of the Intergene study was to investigate the INTERplay between GENEtic susceptibility and environmental factors predisposing to chronic disease. The Intergene study included a questionnaire, a clinical examination and blood measurements. Sample individuals received a letter of information, including an invitation to participate in the study. Written informed consent was required before study inclusion. Screening was centralised to the most inhabited areas, using a mobile test centre (bus) outside the city of Gothenburg, as earlier described by Berg [24].

### Survey information

For self-reported level of PA we used the 4-level Saltin-Grimby Physical Activity Level Scale (SGPALS) [23] which has shown good validity and reliability [6]. The SGPALS has been shown to be related to both the CRF level [25-27] and to CVD-outcomes [28,29].

A single question is used: “How much do you move and exert yourself physically during leisure time? If your activity varies greatly between, for example summer and winter, try to estimate an average. The question concerns the last year”. Four options were given as possible answers, making up the four self-reported physical activity groups. The modifications to modernize the activities of the scale, as outlined below have to our knowledge not been used in exactly the same design earlier in a Swedish cohort. Although the amendment of using computers is logical it hasn’t to our knowledge earlier been used.

1. ***Physically inactive****(I)*: Being almost completely inactive, reading, watching television, watching movies, using computers or doing other sedentary activities, during leisure-time.

2. ***Some****light physical activity (LPA)*: Being physically active for at least four hours/week as riding a bicycle or walking to work, walking with the family, gardening, fishing, table tennis, bowling etc.

3. *Regular physical activity****and training****(moderate PA, MPA)*: Spending time on heavy gardening, running, swimming, playing tennis, badminton, calisthenics and similar activities, for at least 2 to 3 hours/week.

4. *Regular hard physical training for competition sports (vigorous PA, VPA)*: Spending time in running, orienteering, skiing, swimming, soccer, European handball etc. several times per week.

In this study, two percent of the participants reported being physically active at the highest level “hard training or competitive sports” (vigorous PA). This is similar to previous studies, showing that less than four percent of participants report the highest activity level [11,25]. Therefore, we merged group 4 with group 3 to form the moderate-to-vigorous PA group (MVPA), for our study analyses. The definition of physically inactive (formerly described as “Sedentary”), SGPALS 1 above, include those being predominately sedentary and those being active at a very low level. However, the SGPALS does not give information on sitting time or breaks in sitting time.

A problem with the clinical applicability of the SGPALS, may be that since the introduction in 1968, new behavioural concepts and leisure-time activities have been established, including widespread computer use, necessitating updating of the instrument. SGPALS has to our knowledge been translated from Swedish to Norwegian, Danish, Finnish and English.

Questions on educational level, smoking habits, marital status and stress were included in the survey. Smoking was dichotomized into current smoker or non-smoker. Educational level was defined as long or short education, where long education was defined as university level or higher. Marital status was divided into four groups, never married, married/cohabiting, divorced or widow/er. The level of perceived stress at home and work was assessed with a simple question, initially used in the study of men born in 1913 in Gothenburg [30]. In this study self-reported high level of stress was defined as perceived stress during the last year or more at either work or home. This level of stress has been associated with myocardial infarction in previous epidemiological studies [30].

### Measurements and procedure

Participants were told to be fasting for the last four hours prior to the visit at the research site, following standard blood sampling routines. *Weight* and *height* were measured to the nearest centimetre and kilogram, with light clothing and without shoes. *Body Mass Index (BMI)* was calculated, with overweight being defined as a BMI ≥25 kg/m^2^. *Waist circumference* was measured according to international standards, between the lower rib and the iliac crest. High waist circumference was defined as ≥94 centimetres (men) and ≥80 centimetres (women) [31]. *Blood pressure* (in millimetres Hg) was measured twice, for each participant after 5 minutes of rest, using an Omron 711 automatic IS machine, with the subject in the sitting position, with a simultaneous reading of heart rate. An elevated blood pressure was defined as ≥150/90 mmHg. An elevated resting heart rate was defined as ≥70 beats per minute (bpm) [32].

Plasma glucose was analysed using the hexokinase method (Roche Hitachi 917 and Roche ModularP). A high fasting plasma-glucose was defined as ≥6.1 mmol/litre. The cholesterol and triglyceride analyses were made with enzymatic assays and for the high-density lipoproteins (HDL-cholesterol) analyses, dextran sulphate-magnesium precipitation of apo-B containing lipoproteins, were used. LDL cholesterol levels were estimated using the Friedewald equation where applicable (missing n = 58). Unfavourable levels of lipid fractions were defined as, high LDL-cholesterol (≥3.0 mmol/l), low HDL-cholesterol (<1.29 mmol/l women, <1.03 mmol/l men) and triglycerides (≥1.7 mmol/l), as described in earlier epidemiological work [33,34]. During one period there was no blood samples collected (n = 386).

### Statistics

Characteristics for the physical activity groups are presented in the form of means and standard deviations for continuous variables and percents for the categorical variables. To assess the association between the physical activity and elevated cardiovascular risk factors we used a series of logistic regression models where each risk factor was set as dependent binary variable and physical activity and age as independent variables. Analyses with adjustments for educational level affected odds ratios diminutively (data not shown). We also conducted a statistical (contrast analysis) test for differences between the odds ratios of LPA and the inactive group respectively to see if there was a trend of the odds ratios across the physical activity levels. The moderate to vigorous physical activity (MVPA) group was set as reference level in the statistical models above, and odds ratios with the other PA-levels were calculated and presented together with a 95% confidence interval. All analyses were performed using SPSS version 18.0 (SPSS Inc., Chicago, Illinois, USA).

### Ethical review board

This study was approved by the Regional Ethical Review Board, Gothenburg, Sweden, in 2000-12-13 (number 237–00).

## Results

### Level of physical activity in general

Twelve per cent of men and nine per cent of women, respectively, reported being physically inactive, while 59 and 66 % of men and women, respectively, reported LPA, and 29 and 25 % MVPA (Table 1). In Table 1 educational level, smoking habits, stress, marital status and other cardiovascular risk factors are shown for the participants stratified by different physical activity level groups. Individuals reporting MVPA, were twice as likely to report a high educational level and 3–4 times less likely to be smokers, than those reporting being physically inactive.

**Table 1:** Characteristics of the participants according to gender and physical activity level

	**MVPA mean ± SD or % (N)**	**LPA mean ± SD or % (N)**	**Inactive mean ± SD or % (N)**	**All mean ± SD or % (N)**	**Values missing N**
Men	29 (486)	59 (999)	12 (200)	(1685)	
Marital status ^a^	79 (385)	80 (797)	72 (143)	79 (1325)	4
University education	40 (196)	22 (222)	21 (41)	27 (459)	8
Stress ^b^	11 (54)	11 (113)	29 (58)	14 (225)	18
Current smoker	8 (38)	16 (162)	31 (61)	16 (261)	8
Age (years)	48.3 ± 13.5	53.7 ± 12.4	49.2 ± 12.4	51.6 ± 12.9	
Weight (kg)	83 ± 10	85 ± 13	88 ± 15	85 ± 12	
Triglycerides (mmol/l)	1.34 ± 1.15	1.61 ± 0.97	1.97 ± 1.32	1.57 ± 1.09	177
High density lipoprotein (mmol/l)	1.52 ± 0.37	1.45 ± 0.38	1.32 ± 0.34	1.46 ± 0.38	184
Waist circumference (cm)	91 ± 9	96 ± 10	99 ± 12	95 ± 10	52
Resting heart rate (bpm)	61 ± 10	66 ± 11	68 ± 12	65 ± 11	5
BMI (kg/m^2^)	25.8 ± 2.8	26.8 ± 3.5	28.1 ± 4.3	26.7 ± 3.5	
Plasma-glucose (mmol/l)	5.16 ± 0.86	5.43 ± 1.20	5.46 ± 1.27	5.35 ± 1.13	177
Low density lipoprotein (mmol/l)	3.27 ± 0.97	3.39 ± 0.95	3.47 ± 0.88	3.37 ± 0.95	230
Systolic blood pressure (mmHg)	133 ± 18	136 ± 21	132 ± 21	135 ± 20	3
Diastolic blood pressure (mmHg)	81 ± 10	84 ± 11	84 ± 10	83 ± 10	3
Women	25 (469)	66 (1264)	9 (170)	(1903)	
Marital status ^a^	74 (348)	73 (918)	64 (108)	73 (1374)	10
University education	43 (200)	32 (398)	28 (47)	34 (645)	10
Stress ^b^	18 (84)	19 (241)	20 (33)	19 (358)	26
Current smoker	13 (61)	20 (252)	37 (62)	20 (375)	6
Age (years)	46.9 ± 12.7	52.9 ± 13.2	50.0 ± 13.2	51.2 ± 13.3	
Weight (kg)	67 ± 10	70 ± 12	75 ± 15	70 ± 12	1
Triglycerides (mmol/l)	1.05 ± 0.56	1.26 ± 0.68	1.47 ± 0.85	1.22 ± 0.68	207
High density lipoprotein (mmol/l)	1.84 ± 0.44	1.77 ± 0.45	1.62 ± 0.46	1.78 ± 0.45	207
Waist circumference (cm)	79 ± 9	84 ± 11	89 ± 14	83 ± 11	96
Resting heart rate (bpm)	65 ± 11	69 ± 10	70 ± 11	68 ± 11	12
BMI (kg/m^2^)	24.3 ± 3.5	25.9 ± 4.3	27.5 ± 5.6	25.6 ± 4.4	1
Plasma-glucose (mmol/l)	4.91 ± 0.86	5.05 ± 0.89	5.03 ± 0.99	5.01 ± 0.90	207
Low density lipoprotein (mmol/l)	2.94 ± 0.89	3.25 ± 1.01	3.25 ± 0.93	3.18 ± 0.98	222
Systolic blood pressure (mmHg)	124 ± 19	129 ± 23	127 ± 24	128 ± 22	2
Diastolic blood pressure (mmHg)	80 ± 10	82 ± 10	82 ± 11	81 ± 10	2

Data are presented as mean ± standard deviation, or percentage with (N). MVPA, moderate-to-vigorous physical activity, LPA, light physical activity. ^a^ Living together with someone or married. ^b^ Perceiving stress during the last year or more at either work or home. bpm, beats per minute. BMI, body mass index.

### Relationship between level of PA and cardiovascular risk factors

There were higher prevalence of elevated risk factors (body mass index, resting heart rate, waist circumference, plasma-glucose, low level of high density lipoprotein and serum-triglycerides) among those reporting inactive behaviour for both sexes (Table 2), i.e. lower prevalence of risk factors was seen parallel to an increased self-reported PA level. There were only very minor differences in LDL-cholesterol between PA levels. Men and women reporting being inactive (SGPALS level 1), had, on average, nine centimetres greater waist-circumference than individuals reporting MVPA (scoring 3–4 on SGPALS) (Table 1).

**Table 2:** Prevalence of cardiovascular risk factors by gender and physical activity level

	**MVPA % (N)**	**LPA % (N)**	**Inactive % (N)**	**All % (N)**	**Values missing N**
Men	29 (486)	59 (999)	12 (200)	(1685)	
Triglycerides (≥1.7 mmol/l)	20	37	53	34	177
High density lipoprotein (<1.03 mmol/l)	6	11	23	11	184
Waist circumference (≥94 cm)	37	57	67	53	52
Resting heart rate (≥70 bpm)	18	34	37	30	5
Overweight (BMI ≥ 25 kg/m^2^)	58	66	76	65	
Plasma-glucose (≥6.1 mmol/l)	6	13	12	11	177
Low density lipoprotein (≥3.0 mmol/l)	58	65	71	64	230
Hypertension (≥150/90mm/Hg)	13	20	16	17	3
Women	25 (469)	66 (1264)	9 (170)	(1903)	
Triglycerides (≥1.7 mmol/l)	12	20	37	19	207
High density lipoprotein (<1.29 mmol/l)	9	10	19	11	207
Waist circumference (≥80 cm)	43	62	70	58	96
Resting heart rate (≥70 bpm)	30	44	46	40	12
Overweight (BMI ≥ 25 kg/m^2^)	32	52	57	47	1
Plasma-glucose (≥6.1 mmol/l)	2	5	6	4	207
Low density lipoprotein (≥3.0 mmol/l)	45	58	62	55	222
Hypertension (≥150/90mm/Hg)	8	13	15	12	2

MVPA, moderate-to-vigorous physical activity. LPA, light physical activity. bpm, beats per minute. BMI, body mass index.

### Odds ratios for risk factors in relation to the SGPALS

With the most physically active group (MVPA) as reference, men reporting being inactive (SGPALS 1) were more likely to be overweight (OR: 2.19, 95% CI: 1.51-3.19), have a waist circumference ≥94 cm (OR: 3.76, 95 % CI: 2.61-5.43) and have serum-triglycerides ≥1.7 mmol/l (OR 4.5, 95 % CI: 3.08-6.57) Physically inactive women (SGPALS 1), were more likely to be overweight (OR: 2.57, 95 % CI: 1.78-3.73), have a waist circumference ≥80 cm (OR: 2.91, 95% CI: 1.94-4.35) and have serum-triglycerides ≥1.7 mmol/l (OR 3.28, 95 % CI: 2.06-5.22). Furthermore, men reporting inactive behaviour were markedly more likely to report a high level of perceived stress (OR: 3.59, 95 % CI: 2.34-5.49). In fact, odds-ratios for all risk factors were increased in the most inactive group (SGPALS 1), as described in Table 3.

**Table 3:** Age adjusted odds ratios with 95% confidence intervals of physical activity levels for having cardiovascular risk factors in 1685 men and 1903 women

	**MVPA OR**	**Men**		**Women**	
		**LPA OR (95% CI)**	**Inactive OR (95% CI)**	**LPA OR (95% CI)**	**Inactive OR (95% CI)**
Current smoker	1	2.32 (1.59-3.38)	5.24 (3.34-8.20)	1.96 (1.44-2.68)	4.29 (2.82-6.53)
Triglycerides (≥1.7 mmol/l)	1	2.21 (1.68-2.92)	4.50 (3.08-6.57)	1.52 (1.09-2.13)	3.28 (2.06-5.22)
High density lipoprotein ^a^ (mmol/l)	1	1.80 (1.15-2.83)	4.38 (2.59-7.40)	1.24 (0.84-1.85)	2.41 (1.41-4.13)
Waist circumference (≥94 cm men, ≥80 cm women)	1	1.97 (1.55-2.49)	3.76 (2.61-5.43)	1.68 (1.34-2.12)	2.91 (1.94-4.35)
High level of stress at home or work	1	1.28 (0.90-1.83)	3.59 (2.34-5.49)	1.38 (1.04-1.83)	1.25 (0.79-1.98)
Resting heart rate (≥70 bpm)	1	2.24 (1.71-2.94)	2.72 (1.88-3.95)	1.68 (1.33-2.12)	1.96 (1.36-2.82)
Overweight (BMI ≥25kg/m^2^)	1	1.20 (0.96-1.52)	2.19 (1.51-3.19)	1.84 (1.46-2.32)	2.57 (1.78-3.73)
Plasma-glucose (≥6.1 mmol/l)	1	1.73 (1.11-2.70)	2.11 (1.15-3.86)	1.54 (0.74-3.19)	2.45 (0.94-6.42)
Low density lipoprotein (≥3.0 mmol/l)	1	1.25 (0.98-1.60)	1.79 (1.21-2.65)	1.23 (0.96-1.57)	1.79 (1.18-2.72)
Hypertension (≥150/90 mmHg)	1	1.26 (0.92-1.74)	1.22 (0.75-1.99)	1.25 (0.85-1.85)	1.80 (1.03-3.15)

MVPA, moderate-to-vigorous physical activity. LPA, light physical activity. ^a^ <1.03 mmol/l, men, <1.29 mmol/l, women. bpm, beats per minute. BMI, body mass index.

There were significant differences among the odds ratios when comparing them taking into account the model estimated covariance between them and their standard errors for LPA and inactive groups except for hypertension among men and stress among women (data not shown). This analysis show a strong negative association between BMI, weight, resting heart rate, waist circumference and serum-triglycerides and the level of PA for both sexes.

## Discussion

The main finding of the present study was that self-reported physical activity level, according to the SGPALS, was negatively associated with traditional cardiovascular risk factors. The most inactive group (scoring 1 out of a possible 4) has an increased prevalence (odds-ratio) for having pre-defined abnormal levels of several cardiovascular risk factors. While an association between increasing levels of PA and lower levels of most traditional cardiovascular risk factors is well known, our findings may serve as an easy and inexpensive way to identify physically inactive and high risk individuals. Our study also confirms the validity of the modernized SGPALS in the setting of an unselected Swedish population.

While CVD related mortality has, in the last decades, declined in the Western world, CVD still remains a major cause of mortality [3]. Today, the health care system increasingly uses different risk score methods to identify cardiovascular risk patients, for example SCORE as advocated by the European Society of Cardiology [20]. Many existing risk score methods are predictive of future cardiovascular events and/or cardiovascular death [20,21]. However, they do not regularly take into consideration the level of physical activity and/or the maximal oxygen consumption (Vo_2max_), which independently predicts cardiovascular and overall mortality [9]. Indeed, the true CRF in combination with heart rate recovery adds significantly to the predictive power of the Framingham score [9]. Individuals classified by SCORE as low risk, may become medium risk by having a low CRF [35]. Therefore, there is a need for complementary information regarding the CRF level and/or habitual physical activity levels, to increase the yield of the risk stratification in any given individual [35].

In this study we found that individuals reporting being physically inactive during leisure-time, defined as scoring 1 out of 4 on the SGPALS, had elevated cardiovascular risk factors. Physically inactive individuals were four times more likely to be smokers, three times more likely to have an increased waist circumference, a high serum-triglyceride level, and a low serum-HDL level. In addition, individuals reporting being physically inactive, were more likely to have an elevated resting heart rate, having perceived high levels of stress, and elevated plasma-glucose level, and to be overweight (Table 3).

These findings are in line with earlier studies, showing a linear relationship between BMI and CRF [36], as well as the level of PA. It has previously been reported that a higher level of physical activity is associated with lower levels of several cardiovascular risk factors [37], while the lowest levels of PA are being associated with the highest risk [19]. The present definition of SGPALS 1 includes patients being sedentary as well as those reporting a very low leisure-time PA level. This perhaps explains the strong relationships with a multitude of cardiovascular risk factors, as both a low level of PA and sedentary behaviour, independently, are associated with higher cardiovascular risk [38]. The SGPALS indeed seems to capture “the worst of the worst” regarding activity behaviour.

The results of the present study have clinical implications, as the modernized SGPALS may be used as an sole and simple indicator of inactive behaviour (including sedentary and very low level of leisure-time PA), and thus indirectly of cardiovascular risk. The SGPALS has previously been found to be associated with the CRF level of the individual [25-27]. Thus, this scale can be a feasible tool to identify individuals in need of further risk assessment. Physically inactive men also reported increased levels of perceived stress. This is important, since high levels of stress have been related to cardiovascular morbidity, independent of other lifestyle factors [39], supporting the cardiovascular risk assessment potential of SGPALS.

Furthermore, the effectiveness in changing longitudinal outcome by using risk score methods has been reviewed [40], with two studies comparing risk score assessment with placebo showed no benefit, while another study showed an increased use of lipid lowering and antihypertensive drugs, and one study did show a small reduction in systolic BP, using risk assessment. Present risk score tools have probably affected smoking habits [41], while cholesterol levels have been mainly changed by food habits [42]. Whether the use of the SGPALS will affect future health outcome, is of course beyond the scope of this paper. However, the use of a reliable tool for assessment of the PA-level is clinically important, while still underutilized. Therefore, it is also important that the present study validates the modernized SGPALS in the setting of a large Swedish population.

### Limitations and strength

This cross sectional study uses self-reported data concerning the physical activity level and other life style behaviours. Hence there is no objective PA measurement. Furthermore, the study has a response rate of 42 % indicating possible selection bias effect. This has been investigated for the overall Intergene study, showing that participants were more likely to have university education and high income, be married, and to be women of Nordic origin [43]. However, this may theoretically have underestimated the OR for described risk factors. While the SGPALS captures those individuals being sedentary and those having a very low level of leisure-time PA, it gives no information on the actual sitting-time or breaks in sitting-time. A major strength of the study is the population size and its composition of both urban and rural participants from the Region Västra Götaland.

## Conclusions

The present study in a large Swedish cohort, shows that the modernized Saltin-Grimby Physical Activity Level Scale (SGPALS), identifies individuals reporting being predominately sedentary or having a very low leisure-time PA level. Importantly, this physically inactive group was associated with a multitude of elevated cardiovascular risk factors. Accordingly, SGPALS may be useful as a tool in clinical risk assessment, alone or in combination with other risk score methods in everyday health care, to identify physically inactive including sedentary individuals in possible need of PA counselling and further risk assessment.

Hence, we suggest that using the SGPALS in risk assessment, may be suitable as a supportive instrument for health care providers using physical activity on prescription as a preventive and treatment method [44], to improve health care.

## Competing interests

The authors declare that they have no competing interests.

## Authors’ contributions

LR, IJ, AR, LB, DT and MB have made contributions to the conception and design of the study. LR and GL have conducted the statistical analysis. LR drafted the manuscript. IJ and MB have been actively involved in drafting the manuscript. IJ, AR, LB, DT, GG and MB have critically revised the manuscript. All authors read and approved the final manuscript.

## Pre-publication history

The pre-publication history for this paper can be accessed here:

http://www.biomedcentral.com/1471-2458/12/693/prepub

## References

[B1] Caspersen CJ Powell KE Christenson GM Physical activity, exercise, and physical fitness: definitions and distinctions for health-related research Public Health Rep 1985 100 2 126 131 3920711 PMC1424733

[B2] Warburton DE Nicol CW Bredin SS Health benefits of physical activity: the evidence CMAJ 2006 174 6 801 809 10.1503/cmaj.051351 16534088 PMC1402378

[B3] World Health Organization Global health risks: mortality and burden of disease attributable to selected major risks 2009 Geneva: World Health Organization

[B4] Schoenborn CA Adams PF Barnes PM Vickerie JL Schiller JS Health behaviors of adults: United States, 1999–2001 Vital Health Stat 10 2004 219 1 79 15791896

[B5] Sjöström M Oja P Hagströmer M Smith B Bauman A Health-enhancing physical activity across European Union countries: the Eurobarometer study J Public Health 2006 14 5 291 300 10.1007/s10389-006-0031-y

[B6] Aires N Selmer R Thelle D The validity of self-reported leisure time physical activity, and its relationship to serum cholesterol, blood pressure and body mass index. A population based study of 332,182 men and women aged 40–42 years Eur J Epidemiol 2003 18 6 479 485 12908712 10.1023/a:1024682523710

[B7] Ekblom-Bak E Hellenius ML Ekblom O Engstrom LM Ekblom B Independent associations of physical activity and cardiovascular fitness with cardiovascular risk in adults Eur J Cardiovasc Prev Rehabil 2009 17 2 175 180 19809331 10.1097/HJR.0b013e32833254f2

[B8] Lee DC Sui X Ortega FB Kim YS Church TS Winett RA Ekelund U Katzmarzyk PT Blair SN Comparisons of leisure-time physical activity and cardiorespiratory fitness as predictors of all-cause mortality in men and women Br J Sports Med 2010 45 6 504 510 20418526 10.1136/bjsm.2009.066209

[B9] Mora S Redberg RF Cui Y Whiteman MK Flaws JA Sharrett AR Blumenthal RS Ability of exercise testing to predict cardiovascular and all-cause death in asymptomatic women: a 20-year follow-up of the lipid research clinics prevalence study JAMA 2003 290 12 1600 1607 10.1001/jama.290.12.1600 14506119

[B10] Healy GN Matthews CE Dunstan DW Winkler EA Owen N Sedentary time and cardio-metabolic biomarkers in US adults: NHANES 2003–06 Eur Heart J 2011 32 5 590 597 10.1093/eurheartj/ehq451 21224291 PMC3634159

[B11] Jonsdottir IH Rodjer L Hadzibajramovic E Borjesson M Ahlborg G Jr A prospective study of leisure-time physical activity and mental health in Swedish health care workers and social insurance officers Prev Med 2010 51 5 373 377 10.1016/j.ypmed.2010.07.019 20691721

[B12] Bennett JA Winters-Stone K Nail LM Scherer J Definitions of sedentary in physical-activity-intervention trials: a summary of the literature J Aging Phys Act 2006 14 4 456 477 17215562 10.1123/japa.14.4.456

[B13] Healy GN Dunstan DW Salmon J Cerin E Shaw JE Zimmet PZ Owen N Breaks in sedentary time: beneficial associations with metabolic risk Diabetes Care 2008 31 4 661 666 10.2337/dc07-2046 18252901

[B14] Healy GN Wijndaele K Dunstan DW Shaw JE Salmon J Zimmet PZ Owen N Objectively measured sedentary time, physical activity, and metabolic risk: the Australian Diabetes, Obesity and Lifestyle Study (AusDiab) Diabetes Care 2008 31 2 369 371 18000181 10.2337/dc07-1795

[B15] Pate RR O'Neill JR Lobelo F The evolving definition of "sedentary" Exerc Sport Sci Rev 2008 36 4 173 178 10.1097/JES.0b013e3181877d1a 18815485

[B16] Sedentary Behaviour Research Network Letter to the editor: standardized use of the terms "sedentary" and "sedentary behaviours" Appl Physiol Nutr Metab 2012 37 3 540 545 10.1139/h2012-024 22540258

[B17] Katzmarzyk PT Church TS Craig CL Bouchard C Sitting time and mortality from all causes, cardiovascular disease, and cancer Med Sci Sports Exerc 2009 41 5 998 1005 10.1249/MSS.0b013e3181930355 19346988

[B18] Warren TY Barry V Hooker SP Sui X Church TS Blair SN Sedentary behaviors increase risk of cardiovascular disease mortality in men Med Sci Sports Exerc 2009 42 5 879 885 19996993 10.1249/MSS.0b013e3181c3aa7e PMC2857522

[B19] Church TS Earnest CP Skinner JS Blair SN Effects of different doses of physical activity on cardiorespiratory fitness among sedentary, overweight or obese postmenopausal women with elevated blood pressure: a randomized controlled trial JAMA 2007 297 19 2081 2091 10.1001/jama.297.19.2081 17507344

[B20] Conroy RM Pyorala K Fitzgerald AP Sans S Menotti A De Backer G De Bacquer D Ducimetiere P Jousilahti P Keil U Njolstad I Oganov RG Thomsen T Tunstall-Pedoe H Tverdal A Wedel H Whincup P Wilhelmsen L Graham IM Estimation of ten-year risk of fatal cardiovascular disease in Europe: the SCORE project Eur Heart J 2003 24 11 987 1003 10.1016/S0195-668X(03)00114-3 12788299

[B21] Balady GJ Larson MG Vasan RS Leip EP O'Donnell CJ Levy D Usefulness of exercise testing in the prediction of coronary disease risk among asymptomatic persons as a function of the Framingham risk score Circulation 2004 110 14 1920 1925 10.1161/01.CIR.0000143226.40607.71 15451778

[B22] Myers J Kaykha A George S Abella J Zaheer N Lear S Yamazaki T Froelicher V Fitness versus physical activity patterns in predicting mortality in men Am J Med 2004 117 12 912 918 10.1016/j.amjmed.2004.06.047 15629729

[B23] Saltin B Grimby G Physiological analysis of middle-aged and old former athletes Comparison with still active athletes of the same ages Circulation 1968 38 6 1104 1115 10.1161/01.CIR.38.6.1104 5721960

[B24] Berg C Rosengren A Aires N Lappas G Toren K Thelle D Lissner L Trends in overweight and obesity from 1985 to 2002 in Goteborg, West Sweden Int J Obes (Lond) 2005 29 8 916 924 10.1038/sj.ijo.0802964 15852045

[B25] Thune I Njolstad I Lochen ML Forde OH Physical activity improves the metabolic risk profiles in men and women: the Tromso Study Arch Intern Med 1998 158 15 1633 1640 10.1001/archinte.158.15.1633 9701097

[B26] Saltin B Hansen AT, Schnohr P, Rose G Physiological effects of physical conditioning Ischemic Heart Disease: the Strategy of Postponement 1977 Chicago: Year Book Medical Publishers 104 115

[B27] Wilhelmsen L Tibblin G Aurell M Bjure J Ekstrom-Jodal B Grimby G Physical activity, physical fitness and risk of myocardial infarction Adv Cardiol 1976 18 217 230 983849 10.1159/000399526

[B28] Apullan FJ Bourassa MG Tardif JC Fortier A Gayda M Nigam A Usefulness of self-reported leisure-time physical activity to predict long-term survival in patients with coronary heart disease Am J Cardiol 2008 102 4 375 379 10.1016/j.amjcard.2008.03.072 18678290

[B29] Rosengren A Wilhelmsen L Physical activity protects against coronary death and deaths from all causes in middle-aged men. Evidence from a 20-year follow-up of the primary prevention study in Goteborg Ann Epidemiol 1997 7 1 69 75 10.1016/S1047-2797(96)00106-8 9034409

[B30] Rosengren A Tibblin G Wilhelmsen L Self-perceived psychological stress and incidence of coronary artery disease in middle-aged men Am J Cardiol 1991 68 11 1171 1175 10.1016/0002-9149(91)90189-R 1951076

[B31] Carr DB Utzschneider KM Hull RL Kodama K Retzlaff BM Brunzell JD Shofer JB Fish BE Knopp RH Kahn SE Intra-abdominal fat is a major determinant of the National Cholesterol Education Program Adult Treatment Panel III criteria for the metabolic syndrome Diabetes 2004 53 8 2087 2094 10.2337/diabetes.53.8.2087 15277390

[B32] Verrier RL Tan A Heart rate, autonomic markers, and cardiac mortality Hear Rhythm 2009 6 11 Suppl S68 S75 10.1016/j.hrthm.2009.07.017 PMC2776094 19880076

[B33] Graham I Atar D Borch-Johnsen K Boysen G Burell G Cifkova R Dallongeville J De Backer G Ebrahim S Gjelsvik B Herrmann-Lingen C Hoes A Humphries S Knapton M Perk J Priori SG Pyorala K Reiner Z Ruilope L Sans-Menendez S Op Reimer WS Weissberg P Wood D Yarnell J Zamorano JL Walma E Fitzgerald T Cooney MT Dudina A Vahanian A European guidelines on cardiovascular disease prevention in clinical practice: full text. Fourth Joint Task Force of the European Society of Cardiology and other societies on cardiovascular disease prevention in clinical practice (constituted by representatives of nine societies and by invited experts) Eur J Cardiovasc Prev Rehabil 2007 14 Suppl 2 S1 S113 17726407 10.1097/01.hjr.0000277983.23934.c9

[B34] Executive Summary of The Third Report of The National Cholesterol Education Program (NCEP) Expert Panel on Detection, Evaluation, And Treatment of High Blood Cholesterol In Adults (Adult Treatment Panel III) JAMA 2001 285 (19 2486 2497 11368702 10.1001/jama.285.19.2486

[B35] Laukkanen JA Rauramaa R Salonen JT Kurl S The predictive value of cardiorespiratory fitness combined with coronary risk evaluation and the risk of cardiovascular and all-cause death J Intern Med 2007 262 2 263 272 10.1111/j.1365-2796.2007.01807.x 17645594

[B36] Wing RR Jakicic J Neiberg R Lang W Blair SN Cooper L Hill JO Johnson KC Lewis CE Fitness, fatness, and cardiovascular risk factors in type 2 diabetes: look ahead study Med Sci Sports Exerc 2007 39 12 2107 2116 10.1249/mss.0b013e31815614cb 18046181

[B37] Wannamethee SG Shaper AG Physical activity and cardiovascular disease Semin Vasc Med 2002 2 3 257 266 10.1055/s-2002-35400 16222619

[B38] Dunstan DW Thorp AA Healy GN Prolonged sitting: is it a distinct coronary heart disease risk factor? Curr Opin Cardiol 26 5 412 419 21785350 10.1097/HCO.0b013e3283496605

[B39] Rosengren A Hawken S Ounpuu S Sliwa K Zubaid M Almahmeed WA Blackett KN Sitthi-amorn C Sato H Yusuf S Association of psychosocial risk factors with risk of acute myocardial infarction in 11119 cases and 13648 controls from 52 countries (the INTERHEART study): case–control study Lancet 2004 364 9438 953 962 10.1016/S0140-6736(04)17019-0 15364186

[B40] Beswick A Brindle P Risk scoring in the assessment of cardiovascular risk Curr Opin Lipidol 2006 17 4 375 386 10.1097/01.mol.0000236362.56216.44 16832160

[B41] Cooper A Nherera L Calvert N O’Flynn N Turnbull N Robson J Camosso-Stefinovic J Rule C Browne N Ritchie G Stokes T Mannan R Brindle P Gill P Gujral R Hogg M Marshall T Minhas R Pavitt L Reckless J Rutherford A Thorogood M Wood D Clinical Guidelines and Evidence Review for Lipid Modification: cardiovascular risk assessment and the primary and secondary prevention of cardiovascular disease Volume Appendix K - A systematic review of risk scoring methods and clinical decision aids used in the primary prevention of coronary heart disease 2008 London: National Collaborating Centre for Primary Care and Royal College of General Practitioners

[B42] Bjorck L Rosengren A Bennett K Lappas G Capewell S Modelling the decreasing coronary heart disease mortality in Sweden between 1986 and 2002 Eur Heart J 2009 30 9 1046 1056 10.1093/eurheartj/ehn554 19141562

[B43] Strandhagen E Berg C Lissner L Nunez L Rosengren A Toren K Thelle DS Selection bias in a population survey with registry linkage: potential effect on socioeconomic gradient in cardiovascular risk Eur J Epidemiol 2010 25 3 163 172 10.1007/s10654-010-9427-7 20127393

[B44] Swedish Professional Associations for Physical Activity Physical Activity in the Prevention and Treatment of Disease 2010 Swedish: National Institute of Public Health

